# A New Specific Succinate-Glutamate Metabolomic Hallmark in Sdhx-Related Paragangliomas

**DOI:** 10.1371/journal.pone.0080539

**Published:** 2013-11-27

**Authors:** Alessio Imperiale, François-Marie Moussallieh, Frédéric Sebag, Laurent Brunaud, Anne Barlier, Karim Elbayed, Philippe Bachellier, Bernard Goichot, Karel Pacak, Izzie-Jacques Namer, David Taïeb

**Affiliations:** 1 Department of Biophysics and Nuclear Medicine, University Hospitals of Strasbourg, Strasbourg, France; 2 ICube, UMR (Unité Mixte de Recherche) 7357 University of Strasbourg/CNRS (Centre National de la Recherche Scientifique) and FMTS (Fédération de médecine translationnelle de Strasbourg), Faculty of Medicine, Strasbourg, France; 3 Department of Endocrine Surgery, La Timone University Hospital, Aix-Marseille University, Marseille, France; 4 Department of Digestive, Hepato-Biliary and Endocrine Surgery, Brabois University Hospital, Nancy, France; 5 Laboratory of Biochemistry and Molecular Biology, Conception Hospital, Aix-Marseille University, Marseille, France; 6 Department of Visceral Surgery and Transplantation, University Hospitals of Strasbourg, Strasbourg, France; 7 Department of Internal Medicine, Diabetes and Metabolic Disorders, University Hospitals of Strasbourg, Strasbourg, France; 8 Program in Reproductive and Adult Endocrinology, Eunice Kennedy Shriver National Institute of Child Health and Human Development, National Institutes of Health, Bethesda, Maryland, United States of America; 9 Department of Biophysics and Nuclear Medicine, La Timone University Hospital, European Center for Research in Medical Imaging, Aix-Marseille University, Marseille, France; National Research Council of Italy, Italy

## Abstract

Paragangliomas (PGLs) are frequently associated with germline mutations in genes involved in energy metabolism. The purpose of the present study was to assess whether the tumor metabolomic profile of patients with hereditary and apparently sporadic PGLs enables the distinction of different subtypes of tumors. Twenty-eight unrelated patients with a histological diagnosis of PGLs were included in the present study. Twelve had germline mutations in *SDHx* genes (5 *SDHB*, 7 *SDHD*), 6 *VHL*, and 10 were apparently sporadic. Intact tumor samples from these patients (one per patient) were evaluated with ^1^H high-resolution magic angle spinning (HRMAS) NMR spectroscopy. *SDHx*-related tumors were characterized by an increase in succinate levels in comparison to other tumor subtypes (p = 0.0001 vs VHL and p = 0.000003 vs apparently sporadic). Furthermore, we found significantly lower values of glutamate in *SDHx*-related tumors compared to other subtypes (p = 0.0007 vs *VHL* and p = 0.003 vs apparently sporadic). Moreover, *SDHx*-tumors also exhibited lower values of ATP/ADP/AMP (p = 0.01) compared to *VHL*. *VHL* tumors were found to have the highest values of glutathione (GSH) compared to other tumors. Based on 4 metabolites (succinate, glutamate, GSH, and ATP/ADP/AMP), tumors were accurately distinguished from the other ones on both 3- and 2-class PLS-DA models. The present study shows that HRMAS NMR spectroscopy is a very promising method for investigating the metabolomic profile of various PGLs. The present data suggest the existence of a specific succinate-glutamate hallmark of *SDHx* PGLs. The relevance of such a metabolomic hallmark is expected to be very useful in designing novel treatment options as well as improving the diagnosis and follow-up of these tumors, including metastatic ones.

## Introduction

Paragangliomas (PGLs) are neural crest-derived neuroendocrine neoplasms arising from chromaffin cells that are located in the adrenal medulla (also called pheochromocytomas, PHEOs) or aligned along the para-aortic sympathetic system or from head and neck or thoracic paraganglia, which often act as chemoreceptors. Although most PGLs arise sporadically, PGL susceptibility genes have been identified in approximately one-third of cases and in more than 90% of patients with multifocal tumors [1], [2].

Hereditary PGLs are frequently associated with germline mutations in one of the succinate dehydrogenase subunits genes (collectively named *SDHx*-related tumors), often in the absence of positive family history or even with negative biochemistry [3]. This is in contrast to other hereditary PHEOs/PGLs, in which the family history is usually very well known and therefore, PHEOs/PGLs related to these syndromes can be diagnosed early and often properly and successfully treated.

Moreover, *SDHx* tumors are considered to be more aggressive, particularly those with *SDHB* mutations. Currently there is no cure, and current therapeutic options are either suboptimal or short-lasting despite a good initial response [4]. Therefore, any new information that could bring new insight into the pathogenesis of these tumors, their metabolic activities, and proper monitoring, including early assessment of their therapeutic responses, is very desirable.

SDH (also called respiratory complex II) is an iron-sulfur cluster-containing protein composed of 4 subunits that participates in the tricarboxylic acid (TCA) cycle and electron transport chain (ETC). SDH mediates the transfer of two electrons to CoQ during the oxidation of succinate to fumarate. Inactivation of SDH leads to the accumulation of succinate. The accumulation of specific metabolites has been illustrated in different tumor models with inherited and acquired alterations in enzymes of the TCA cycle, such as fumarate in cases of fumarate hydroxylase gene mutations [5] and 2-hydroxyglutarate (2HG) in mutations in one of the 2 isocitrate dehydrogenase genes (*IDH1/2*) [6]. The accumulation of these oncometabolites inhibits 2-oxoglutarate (2OG)-dependent dioxygenases that include EglN (also called PHD) family members, methylcytosine dioxygenases and JmjC domain-containing histone demethylases, which induces both pseudohypoxic and hypermethylated phenotypes [7]–[10]. These findings have important implications for our understanding of tumorigenesis via pseudohypoxia, alteration of epigenetic homeostasis and alteration of the cellular redox state.

Simultaneous detection of several metabolites (also called metabolomics) is a fast-growing field, promising to improve our understanding of cell biology and relating the genetics and epigenetics to tumor phenotypes. The metabolites (molecular weight <1 kDa) can be detected *in vitro* by gas chromatography-mass spectrometry (GCMS), liquid chromatography-mass spectrometry (LCMS), and nuclear magnetic resonance (NMR) spectroscopy [11]. Magnetic resonance spectroscopy also enables *in vivo* assessment of metabolites (such as 2HG) in the setting of brain tumors [12]. NMR-based methods are less sensitive but more reproducible than LCMS for the quantitative assessment of metabolites.

In recent years, ^1^H high-resolution magic angle spinning (HRMAS) NMR spectroscopy has been introduced for *ex vivo* characterization of small intact tissue samples (solid-state NMR). This technique also offers several advantages, such as the simplicity of sample preparation, the intra- and inter-laboratory reproducibility, the relatively low cost to perform the technique, and the availability of major metabolite databases [13]. HRMAS NMR spectroscopy has been suggested as a promising tool in the diagnosis and characterization of cancer.

The objective of the present study was to find out whether there is a specific metabolomic profile in sympathetic *SDHx* and other hereditary or apparently sporadic PHEOs/PGLs.

## Materials and Methods

### Ethics Statement

Data was acquired under regular clinical care conditions, with Ethics Committee approval obtained for the use of these data for scientific purposes at Strasbourg, Marseille, and Nancy University Hospitals. Written informed consent was obtained from all patients included in the present study.

### Tissue samples

Twenty-eight specimens of sympathetic PGLs/PHEOs from 28 unrelated patients who fulfilled the following criteria were included:

1. Histological diagnosis of PGL/PHEO.
2. Absence of distant metastases within the first year following surgery.
3. Genetic screening for germline mutations in the *SDHB/C/D, SDHAF2* (including large gene rearrangements of all the *SDH* genes), *VHL* (including large gene rearrangements), *RET, TMEM127*, and *MAX* genes.
4. Tissue specimens collected just after tumor removal and snap-frozen in liquid nitrogen before storage at −80°C.

The tumors were obtained from 3 different institutions in France (Strasbourg, Marseille, and Nancy University Hospitals) and were distributed as follows: 10 sporadic (9 PHEOs, 1 sympathetic PGL), 5 *SDHB* (2 PHEOs, 3 sympathetic PGLs), 7 *SDHD* (1 PHEO, 6 sympathetic PGLs), and 6 *VHL* (3 PHEOs, 3 sympathetic PGLs).

### Tissue sample preparation for HRMAS NMR

The amount of tissue used for HRMAS analysis ranged from 15 to 20 mg. For each sample, the percentage of tumor cells in the total sample of cells and the percentage of necrosis with regard to the total surface were calculated based on frozen sections using a mirror sample stored in the tissue bank. Samples containing at least 30% tumor cells and less than 50% necrosis were used for the study. Each tissue sample was placed in a 30 µL disposable insert. 10 µL of D_2_O were added to the rotor to provide a lock frequency for the NMR spectrometer. The exact weight of the sample used was determined by weighing the empty insert and the insert containing the tissue sample. The insert was stored at −80°C and placed in a 4-mm ZrO_2_ rotor just before the HRMAS analysis.

### HRMAS NMR data acquisition, spectra processing, and metabolite quantification

1D HRMAS NMR spectra were recorded on a Bruker Avance III 500 spectrometer operating at a proton frequency of 500.13 MHz, installed at the Pathological Department of Strasbourg University Hospitals. A one-dimensional (1D) proton spectrum using a Carr–Purcell–Meiboom–Gill (CPMG) pulse sequence [14] and 1024 transients was acquired for each serum sample. Free induction decays were multiplied by an exponential window function of 0.3 Hz prior to Fourier transformation and were corrected for phase and baseline distortions using TopSpin 2.1 (Bruker GmbH, Germany). The chemical shift was referenced to the peak of the methyl proton of L-lactate at 1.33 ppm. In order to confirm resonance assignments, two-dimensional (2D) homonuclear and heteronuclear experiments were also recorded immediately after the end of 1D spectra acquisition. Because the duration of these experiments is long and significant tissue degradation occurs during NMR acquisition, only a few representative samples were analyzed by 2D experiments. A description of the HRMAS NMR data acquisition and spectra processing has been previously detailed [13].

In the present study, we focused on four selected metabolites with a potential key role in tumoral pathophysiology. Accordingly, succinate, glutamate, glutathione, and energy phosphorylated compounds were precisely identified and quantified. Catecholamines were also measured. The quantification procedure was based on the pulse length-based concentration measurement (PULCON) as previously described [13]. Spectra were normalized according to each sample weight and calibrated using the signal intensity of a 19.3 nmol reference solution of lactate, scanned under the same analytical conditions. The peak integral corresponding to each metabolite's region was normalized to the integral of the entire spectrum within the range of 8.65–1 ppm. Quantification results were expressed as nmol/mg of tissue. Metabolites were assigned using standard metabolite chemical shift tables available in the literature [15], [16].

Succinate concentrations in tissue samples were estimated by integrating the area comprised between 2.39 ppm and 2.43 ppm. The spectral complex previously assigned to ATP/ADP/AMP [17] was identified on our spectra within the range of 6.07–6.11 and thus integrated to quantify the metabolite amount. The glutathione and glutamate amounts were measured from the integral of NMR spectra comprised by 2.93–2.98 ppm and 2.32–2.38 ppm, respectively. To measure epinephrine concentrations in tissue samples, the signal resulting from the N-methyl radical selected at 2.75 ppm was considered. The 3,4-dihydroxybenzene groups of both epinephrine and norepinephrine generate a spectral complex between about 6.85 and 6.98 ppm. The integral of the region corresponding to the ^1^H in position no. 5 of the aromatic ring (IUPAC nomenclature) was selected to quantify the amount of epinephrine plus norepinephrine in each tissue sample. Finally, the norepinephrine concentration was obtained by subtracting epinephrine from the sum of epinephrine and norepinephrine. The above approach has been previously tested and confirmed (data not shown) by the quantification NMR analysis of epinephrine and norepinephrine standard solutions first separated and subsequently mixed (1/1, v/v). Dopamine could contribute to peaks in the region between 6.85 and 6.98 ppm. However, no triplets at 2.85 ppm and 3.22 ppm, which represent the spectral signature of dopamine, were detected on HRMAS NMR spectra, suggesting an undetectable amount of dopamine in the analyzed tissue.

### Statistical analysis

As widely suggested [18], [19], a combination of principal component analysis (PCA) and partial least square discriminant analysis (PLS-DA) was adopted. PCA and PLS-DA analysis were done including only succinate, glutamate, glutathione, and energy phosphorylated compounds.

A PCA was performed to evaluate the quality of the data quickly and to identify possible outliers [20]. Then a PLS-DA was employed to optimize the separation between groups and to classify the samples in each of the following 3- or 2-group models: 1. Sporadic vs. *SDHx* vs. *VHL*, 2. Sporadic vs. *SDHx*, 3. Sporadic vs. *VHL*, 4. *SDHx* vs. *VHL*. Cross-validation was used in each PLS-DA model to determine the number of components and to avoid overfitting the data because of the small number of samples. An extensive cross-validation embedded in a Monte-Carlo resampling approach was used during the construction of the model in order to build a confusion matrix that allowed a direct visualization of the performances of the model in term of classification power (sensitivity (Se), specificity (Sp), positive predictive value (PPV), negative predictive value (NPV)) [21], [22]. The non-parametric Mann-Whitney U-test (MWU-test) was utilized for the comparison of metabolite amounts between groups. The results of tissular metabolite concentrations are expressed as medians and ranges. The relationship among the selected metabolites was assessed by the Spearman nonparametric regression coefficient (R).

SIMCA P (version 11.0, Umetrics AB, Umeå, Sweden) and STATISTICA 7 (STATSOFT; www.statsoft.com) packages were used for statistical data analysis. A *p* value less than 0.05 was considered statistically significant.

## Results

### General findings

**Figure 1:**
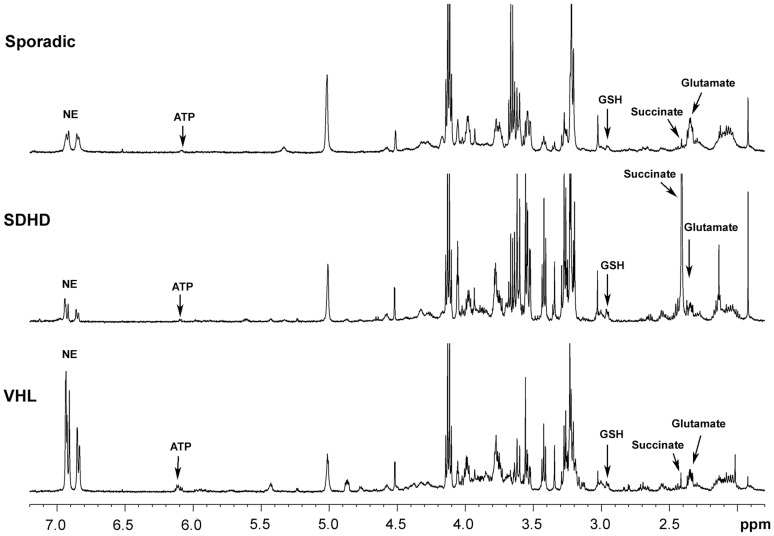
Representative 1D ^1^H-HRMAS spectra of sporadic, *SDHx*-, and VHL-related PGLs. The partial metabolite assignment is indicated. The metabolic contents of each spectra are directly comparable, since the intensity of each spectrum has been normalized with respect to the weight of the analyzed sample.

Representative 1D HRMAS CPMG spectra of sporadic, *SDHD*-, and VHL-related PGLs are presented in Figure 1. Metabolites such as succinate, glutamate, glutathione (GSH), and energy phosphorylated compounds (ATP/ADP/AMP) were identified in 1D spectra, with variable signal intensity according to the genetic background.

**Figure 2:**
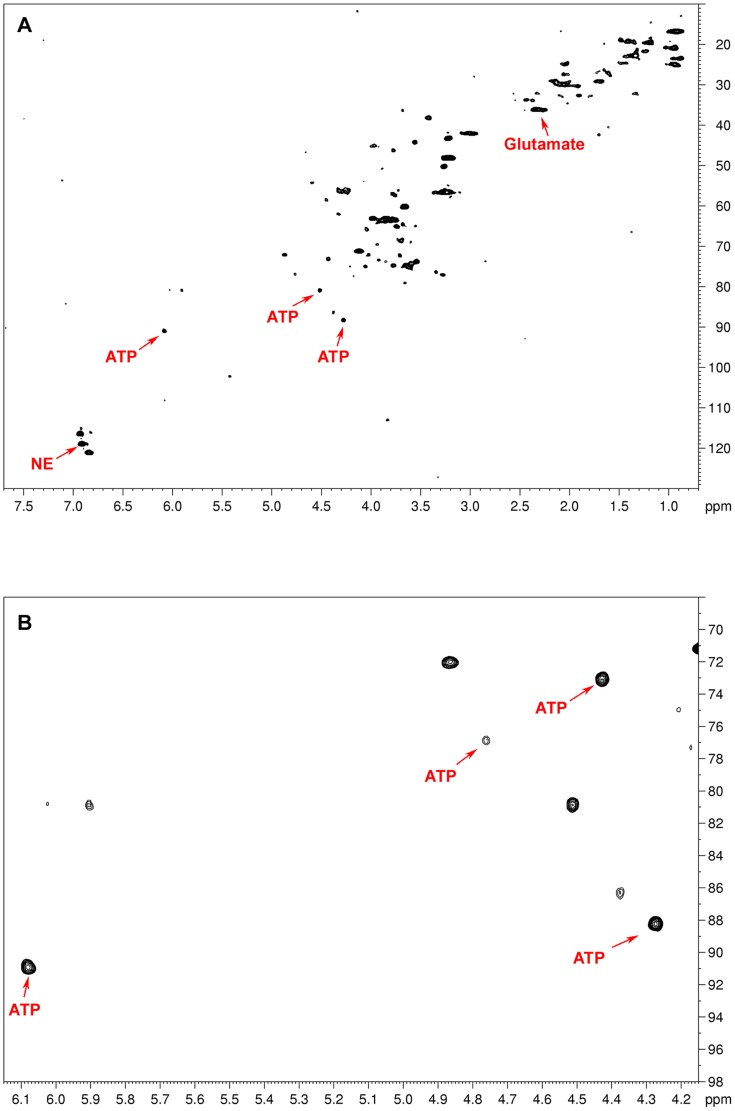
Representative 2D ^1^H-^13^C HSQC HRMAS NMR spectra (A), zoomed in on the ATP region (B), obtained from a sample of a VHL-related abdominal PGL. The 2D NMR experiment confirms the presence of ATP in tumoral tissue as suggested by the 1D spectra of the same patient (Figure 1C). Glutamate and norepinephrine assignments are also indicated. Because of their low concentration in the tissular sample, both succinate and glutathione are not clearly visible in the whole 2D spectrum (7.7-0.7 ppm in ^1^H dimension) without focusing on their characteristic regions.

In all the examined tumor samples, no significant spectra overlap that prevented quantification was found. The quality of the examined samples was indirectly proven by the lack of signals arising from both lactate and fatty acids, which are usually considered hallmarks of tissular necrosis. 2D NMR acquisitions were used to better characterize the metabolic substrate appearing on our 1D spectra within the range of 6.07–6.11 ppm and previously assigned to ATP/ADP/AMP (Fig. 2). In particular, the results of 2D HRMAS acquisition from one VHL-related PGL were compared to the widely available NMR spectroscopy database [16], [23]. Our 2D HSQC spectrum was almost superimposable to the known reference of ATP. Nevertheless, a slight chemical shift difference of 0.06 ppm related to the carbon atom linking the ribosyl ring and the aminopurinine may generate confusion with inosine triphosphate (ITP). This difference could be explained by the intratissular pH variability in our samples.

All NMR spectra from the *SDHx*-related tumors showed very high levels of succinate compared to sporadic and VHL-related tumors. On the other hand, apparently sporadic tumors were characterized by high values of glutamate compared to *SDHx*-related PGLs/PHEOs. Finally, VHL-related tumors exhibited more pronounced ATP/ADP/AMP signals. GSH was predominant in *SDHx*- and VHL-related tumors.

### Three-class model

**Figure 3:**
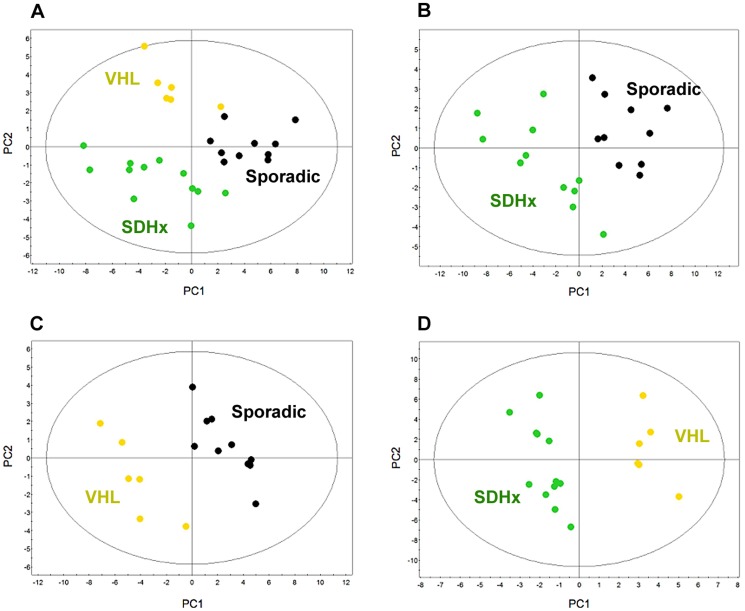
Results of two-component PLS-DA models built on succinate, glutamate, glutathione, and energy phosphorylated compounds (ATP/ADP/AMP) according to the patients' genotypes: A) 3-class model including apparently sporadic (black dots), *SDHx*- (green dots), and VHL-related tumors (yellow dots); B) 2-class model including apparently sporadic and *SDHx*-related tumors; C) 2-class model including apparently sporadic and VHL-related tumors; D) 2-class model including *SDHx*- and VHL-related tumors. A clear distinction between the different classes of tumors is shown in each model.

A multivariate two-component PLS-DA based on succinate, glutamate, GSH, and energy phosphorylated compounds (ATP/ADP/AMP) was first generated including patients with apparently sporadic, *SDHx*-, and VHL-related PGLs/PHEOs. The score plot of the PLS-DA model (Fig. 3A) showed a clear distinction between these three classes of tumors. According to this model, all 12 patients with *SDHx*-related PGLs/PHEOs were correctly identified. Moreover, eight of ten (80%) and five of six (83%) patients with respectively apparently sporadic and VHL-related PGLs/PHEOs were also accurately classified. To detect patients with germline mutations (*SDHx* and *VHL*), the Se and Sp of the established model were respectively 94% and 80%. PPV, NPV, and global accuracy were all 89%.

In the *SDHx*-related tumor cohort, PHEOs exhibited higher values of energy-phosphorylated compounds (ATP/ADP/AMP) compared to PGLs (p = 0.04). Succinate, GSH, and glutamate were statistically equivalent regardless of the *SDHx* tumor location. In contrast, VHL-related PHEOs had higher GSH levels than PGLs (p = 0.04). Considering all tissue samples, a significant negative correlation was shown between succinate and glutamate (R = 0.52), glutamate and energy phosphorylated compounds (ATP/ADP/AMP) (R = 0.54), and succinate and GSH (R = 0.51).

### Two-class models

Three multivariate PLS-DA models based on succinate, glutamate, GSH, and energy phosphorylated compounds (ATP/ADP/AMP) were built to compare: (1) apparently sporadic to *SDHx*- (Fig. 3B), (2) apparently sporadic to VHL- (Fig. 3C), and (3) *SDHx*- to VHL-related tumors (Fig. 3D). The score plot of each PLS-DA model showed a very clear delineation of the single classes. The results of the MWU-test are detailed in Table 1.

**Table 1:** Median values and ranges of succinate, glutathione, glutamate, and ATP/ADP/AMP levels measured by 1D ^1^H-HRMAS spectroscopy in intact tissue samples of 28 apparently sporadic, *SDHx*, and VHL-related PHEOs/PGLs. Quantification results are expressed as nmol/mg of tissue.

	Sporadic	SDHx	VHL	*P* Sporadic vs SDHx	*P* Sporadic vs VHL	*P* SDHx vs VHL
Succinate	0.03 (0–0.06)	3,6 (0.9–9.07)	0.06 (0.03–0.12)	0.000003	0.02	0.0001
Glutathione	0.04 (0.03–0.22)	0.15 (0.05–0.39)	0.32 (0.17–0.43)	0.01	0.003	0.08
Glutamate	1.36 (0.23–2.55)	0.67 (0.13–1.52)	1.49 (0.88–2.18)	0.003	1	0.0007
ATP/ADP/AMP	0.10 (0.03–0.18)	0.05 (0–0.21)	0.16 (0.06–0.29)	0.1	0.1	0.01

When comparing apparently sporadic to *SDHx*-related tumors, the model correctly classified 9 of 10 apparently sporadic cases and 11 of 12 *SDHx*. The model identified *SDHx*-related tumors with a Se of 92%, a Sp of 90%, a PPV of 92%, a NPV of 90%, and a global accuracy of 91%. The model did not correctly identify only one *SDHD*-related PGL. Interestingly, it was easily recognized by visual inspection of the spectra because of a pronounced succinate peak.

In the model comparing apparently sporadic and VHL-related tumors, all cases were correctly classified. The VHL group exhibited a significant increase in both succinate and GSH levels.

Finally, *SDHx*- and VHL-related PGLs/PHEOs were compared. The model was able to correctly classify all the tumor samples. Succinate remains highly discriminant between the 2 subgroups. No difference in the succinate amount was shown between *SDHB* and *SDHD* subtypes, as well as for the remaining selected metabolites. VHL-related tumors showed a more abundant content of energy phosphorylated compounds (ATP/ADP/AMP). Interestingly, despite a greater amount of GSH in VHL vs. *SDHx* tumors, this parameter did not reach statistical significance (p = 0.08).

In apparently sporadic PGLs/PHEOs, glutamate and energy phosphorylated compounds (ATP/ADP/AMP) (R = 0.71) as well as succinate and GSH (R = 0.68) in *SDHx*-related tumors were statistically correlated.

Epinephrine was not measurable in any VHL-related tumors and in only one of 12 *SDHx* samples (1.98 nmol/ml). Only 2 of 10 apparently sporadic tumors showed an epinephrine peak in the NMR spectra (0.93 and 1.04 nmol/ml). On the other hand, norepinephrine was detected in 9 of 10 apparently sporadic tumors (median, 0.90 nmol/ml; range, 0–4.60 nmol/ml), in 9 of 12 *SDHx*-related PHEOs/PGLs (median, 0.87 nmol/ml; range, 0–2.40 nmol/ml), and in all of the 6 VHL-related PHEOs/PGLs (median, 1.80 nmol/ml; range, 0.26–4.53 nmol/ml). No statistical difference was observed between VHL and *SDHx* PHEOs/PGLs despite higher values of norepinephrine in VHL tumors.

## Discussion

In the present study, we analyzed 28 sympathetic PGLs with different genetic backgrounds (10 sporadic, 6 VHL, 12 SDHx). We did not include *RET*- or NF1-related tumors because these are almost always associated with other syndromic manifestations that enable physicians to easily diagnose them.

In the present study we show that HRMAS NMR-based metabolomics should be considered as a new reliable tool for *ex vivo* characterization of sympathetic PGLs. The present data suggests the existence of a specific succinate-glutamate hallmark of *SDHx* sympathetic PGLs. Based on 4 metabolites (succinate, glutamate, GSH, and ATP/ADP/AMP), tumors were accurately distinguished from the other ones on both 3- and 2-class PLS-DA models. This is an extension of the results published by Rao et al., who identified an *SDHx* signature (increased succinate, decreased ATP/ADP/AMP) but with some overlaps between apparently sporadic and VHL tumors for the other parameters [17].

Until the present, only a few studies employing HRMAS NMR spectroscopy have been published for metabolic profiling of PGLs/PHEOs [17], [24]. An increase in succinate accumulation and a decrease in ATP/ADP/AMP accumulation were observed in *SDHx*-related PGLs when compared to sporadic PGLs and PGLs of other genotypes [17]. Interestingly, despite methodological differences, we found similar results. Compared to the previous study [17], in which liquid NMR spectroscopy was used to analyze homogenized and centrifuged tissue preparation, our analysis was done on intact tissue samples. This approach may be considered less sensitive compared to liquid-state NMR spectroscopy but also leads to good quality spectra. Furthermore, it has several advantages, such as avoiding technical procedures for tissue preparation prior to the analysis and allowing refreeze and storage of tissue samples for additional analyzes such as pathological analysis in cases of result discrepancies. Small changes (0.1 ppm) in chemical shift in the peak areas assigned to the ATP/ADP/AMP between the 2 studies could be attributed to pH differences (perchloric acid extracts for Rao et al) [17].

We also found lower values of ATP/ADP/AMP in *SDHx*-related tumors in comparison to VHL tumors, probably because increased glycolysis preferentially occurs in VHL tumors [17], [25].

We have added greater levels of discrimination by using additional metabolites besides succinate and ATP/ADP/AMP peaks. We found that apparently sporadic tumors were characterized by a low level of reduced GSH compared to VHL tumors (p = 0.01) and *SDHx*-related tumors (p = 0.003). There was a trend towards statistical differences between *SDHx*- and VHL-related tumors (p = 0.08), but the sample size was too small to achieve statistical significance. GSH is the most prevalent non-protein thiol in animal cells. Its high redox potential renders GSH both a potent antioxidant and a convenient cofactor for enzymatic reactions that require readily available electron pairs. GSH appears to be a sensitive indicator of the ability to resist toxic challenge. It has previously been shown that sporadic tumors may also exhibit reduction of mitochondrial respiration [26]. The low GSH peak in these tumors could be related to several cell events, such as inhibition of the ETC that is needed for GSH regeneration via NADPH synthesis and/or an increase in ROS production.

Interestingly, *SDHx*-related tumors were also characterized by low glutamate levels in comparison to apparently sporadic (p = 0.003) and VHL tumors (p = 0.0007). This finding is new and provides critical information for accurate classification of tumors. Intracellular glutamate is mainly produced by the oxidative deamination of glutamine. Interestingly, *IDH1/2* mutant cells are also characterized by lower levels of glutamate with respect to their wild type counterparts [27]. It is also possible that lower glutamate availability in *SDHx*-deficient tumors leads to the generation of perooxidant conditions that promote carcinogenesis. There is no clear explanation for the low glutamate level in *SDHx*-related tumors in comparison to other subtypes. It could be related to increased consumption of glutamate for further biosynthetic pathways or decreased glutamate biosynthesis via modulation of the activity of glutaminase or glutamate dehydrogenase.

In contrast, increased GSH levels in VHL-tumors may be related to increased activity of the pentose phosphate pathway in response to enhanced oxidative stress.

Our 3-class model enabled us to accurately predict the presence of *SDHx* or *VHL* germline mutations with the following performances: Se 94%, Sp 80%, and PPV/NPV 89%. The presence of a succinate peak alone easily distinguishes *SDHx*-related tumors from the other ones. The 2-class model based on succinate/GSH could be used to distinguish VHL from apparently sporadic tumors (global accuracy of 100%). Although our results are promising, they should be validated in a large sample size and independent cohort. This would help build a reliable probability predictive model that may be used in clinics to guide genetic testing and provide functional information about mutations of equivocal pathogenicity (uncertain variants).

Immunohistochemical studies can also be used as a screening method for guiding genetic testing. The absence of *SDHB* immunoexpression is indicative of a germline mutation in one of the *SDHx* genes [28], [29]. Additionally, a subset of these tumors may also be immunohistochemically negative for *SDHA*, which is highly suggestive of the presence of an *SDHA* mutation [30]. Compared to other methods, metabolomic approaches provide several practical advantages, such as the simplicity of sample preparation, reproducibility, low cost for sample analysis, and simultaneous measurement of several metabolites. Thus, they may enable global metabolomic profiling of tumors with a distinction of different tumor subtypes. Metabotyping various tumors, including PGLs, might indeed identify new oncometabolites for their future diagnostic and therapeutic options.

The present study further supports the existence of different secretory phenotypes in PGLs according to their hereditary background. In our series, epinephrine was not measurable in any VHL-related tumors and in only one of the 12 *SDHx* samples. By contrast, norepinephrine was detected in 9 out of 12 *SDHx*-related tumors and in all of the 6 examined VHL-related PHEOs/PGLs. The lack of epinephrine secretion in VHL- and *SDHx*-related PGLs was previously reported [31]–[33] and linked to reduced or absent expression of phenylethanolamine-N-methyltransferase (PNMT). PNMT catalyzes the last step in catecholamine biosynthesis, the conversion of norepinephrine to epinephrine. *SDHx*-related tumors exhibit an immature catecholamine secretory profile by the absence of epinephrine, possibly explained by hypermethylation of the PMNT promoter [34]. These tumors accumulate succinate, which inhibits 2-oxoglutarate-dependent histone and DNA demethylase enzymes, resulting in epigenetic modifications [8]. These findings further emphasize the interplay between the Krebs cycle, oxidative phosphorylation, and epigenetic events [35], [36].

We acknowledge several limitations to the present study: a) the absence of carriers of mutations in the *TMEM127*/*SDHAF2*/*MAX*/*SDHA* genes; and b) the absence of screening of tumors for somatic mutations, mainly in apparently sporadic tumors, that could potentially occur and influence the presented tumor metabolomic profiles. We also have to admit that the high proportion of hereditary PGLs in our series is related to a selection bias. Nevertheless, the present study provides new data about specific genotype-metabolomic phenotype of *SDHx*-related PGLs compared to VHL-related and apparently sporadic counterparts.

The present study advances our knowledge of the pathogenesis of PGLs and also provides important and promising information about the classification of these tumors. It is expected that in the near future, various metabolomic approaches will reveal new diagnostic and therapeutic targets. A large-scale study comparing PGL clinical and imaging phenotypes, genotypes, somatic mutations, gene expression levels, DNA methylation phenotypes, protein spectra, and metabolomic profiles will have a great impact on our understanding of how they function and contribute to the development of various PGLs, resulting in the most optimal early and accurate diagnosis, treatment, and outcome.
